# Artificial intelligence in general practice in Germany: an online survey of current use, perceived benefits, barriers, and future needs

**DOI:** 10.3399/BJGPO.2025.0166

**Published:** 2025-12-19

**Authors:** Anne Werner, Stefanie Fischer, Markus Bleckwenn, Anne Schrimpf

**Affiliations:** 1 Institute for General Practice, Faculty of Medicine, University of Leipzig, Leipzig, Germany

**Keywords:** artificial intelligence, primary health care, digital health, general practitioners

## Abstract

**Background:**

Artificial intelligence (AI) is increasingly recognised as a transformative tool in health care. However, despite these prospects, the adoption of AI in primary care remains limited in Germany owing to various concerns.

**Aim:**

To investigate current AI use, perceived barriers, and training needs among GPs. It also aims to compare attitudes of current users and non-users of AI technologies.

**Design & setting:**

This was a cross-sectional study of practising GPs in the Free State of Saxony, Germany; participants received an invitation to participate in an online survey between November 2024 and January 2025.

**Method:**

In total, 1620 GPs received an invitation and two additional reminders to complete the survey via REDCap.

**Results:**

A total of 154 GPs completed the questionnaire, of whom *n* = 70 currently use AI in practice, predominantly for therapy (*n* = 51, 72.9%) and speech recognition or transcription (*n* = 47, 67.1%). The biggest barrier to AI implementation was a lack of knowledge about suitable AI-based applications and of how AI works. Compared with AI users, non-users expressed greater legal concerns, more concerns about patient acceptance, and less familiarity with suitable AI applications; they also perceived AI as less relevant to their daily work. To reduce barriers, participants emphasised a favourable cost–benefit ratio of AI applications and facilitating seamless integration into practice software. The majority of responders (*n* = 83 out of 149, 55.7%) expressed a need for further training on using AI. GPs emphasised the importance of maintaining the interpersonal relationship in health care.

**Conclusion:**

Our study underscores the need for targeted training programmes that address GPs’ specific needs and concerns.

## How this fits in

AI is increasingly recognised as a promising tool in health care, but its use in general practice remains limited. Previous research has largely focused on potential applications rather than current adoption and attitudes of GPs, especially in the context of routine care. This study provides insights on how GPs in Saxony, Germany, are using AI, the barriers they face, and what support they need. The findings highlight a clear need for targeted training and practical integration strategies to support AI adoption in general practice.

## Introduction

The integration of AI into health care has attracted considerable attention in recent years. By using advanced algorithms and large datasets to identify patterns and generate data-driven decisions, AI is increasingly regarded as a transformative force in the healthcare sector.^1,2^

Recently, the German Association of General Practitioners published a position paper highlighting the potential of integrating AI into primary care to enhance both the efficiency and quality of health care, while alleviating the workload of medical and non-medical staff.^3^ The main areas include the automation of administrative tasks — such as managing patient records, organising clinical findings, scheduling appointments, and processing billing — and improving the speed and accuracy of diagnoses. Furthermore, AI may support patient communication, for example, by offering real-time translation during consultations or answering basic health-related queries outside of consultation hours.^3^

Despite these prospects, the adoption of AI in primary care remains limited in Germany, and many GPs continue to express reservations about its use.^4^ A range of barriers to implementation has been identified, including concerns about data security, legal responsibility, lack of technical infrastructure, insufficient training, and cost-effectiveness.^5–9^ As AI technologies continue to advance, ongoing assessment of physician attitudes is necessary. Therefore, the present study aimed to investigate the current use of AI in general practices in Germany. Through a structured questionnaire distributed to practising GPs, we sought to assess current experiences, identify perceived barriers, and explore potential areas for AI integration. Additionally, we compared attitudes towards AI use between GPs who currently use AI in their practice and those who do not. Furthermore, the study aimed to gain insight into GPs’ training needs and their attitudes towards the use of AI technologies in routine clinical practice.

## Method

### Recruitment procedure

The data were collected in the Free State of Saxony, Germany, between November 2024 and January 2025. All GPs actively practising in Saxony with an available email address were eligible to participate; those without an email address were excluded. The email addresses were obtained from the Association of Statutory Health Insurance Physicians Saxony (Kassenärztliche Vereinigung Sachsen). Of the 1962 GPs working in the Free State of Saxony in 2024 (1764 established and the remainder employed with specialist qualifications),^10^ 1620 had published an email address and therefore formed the sampling frame. Each received a personalised invitation email in early November 2024 with study information, a link to the online questionnaire, and a unique access token to prevent duplicate participation. Consent was obtained electronically via an ‘I agree’ button before starting the survey. Non-responders were sent reminders at the end of November 2024 and again in January 2025, following recommended survey methodology.^11^ Invalid email addresses (‘bounces’) were documented and excluded from the denominator when calculating response rates. Partial responses were treated as non-responses.

### Questionnaire

The questionnaire was self-developed in the Institute for General Practice, Leipzig University, by an interdisciplinary research team (psychologist, physicians, medical and public health scientists) and underwent a think-aloud pre-testing process with three GPs. The translated questionnaire can be found in Supplementary material S1**.** The response formats were choice answers, 5-point Likert scales, and free-text entries. All structured questionnaire items were mandatory, except for optional free-text fields where responses were not required. For the online survey, the software REDCap (Research Electronic Data Capture; Vanderbilt University) was used, hosted on a secure server of the Leipzig University Computer Centre. The completion of the online survey took 5–10 minutes.

### Statistical analyses

All statistical analyses were carried out using SPSS Statistics (version 29) with a two-sided α-level of 0.05. For descriptive statistics, missing values in single variables were considered by presenting frequencies as % (*n*/nvalid). As all structured questionnaire items were mandatory, nvalid is identical to the total sample size (*n*). Continuous variables were presented as mean (M)±standard deviation (SD).

Differences in age and number of years worked as a GP after specialist qualification between participants who use AI in their practice and those who do not were examined using univariate analyses of variance. Differences in categorical demographic variables, as well as in perceived barriers to AI use, were analysed using χ^2^ tests or Fisher’s exact tests, as appropriate. Effect sizes were estimated and reported using Cramér’s V.

Free-text entries were coded in major and subcategories by two authors of this study (SF, AS). The assignments were compared and differences in coding were discussed until inter-coder agreement was reached for each discrepancy. As free-text responses were optional, we always reported nvalid for these items to reflect the number of participants who provided a response.

## Results

### Sample characteristics

Of 1620 contacted GPs, a total of 154 participants completed the questionnaire (response rate 9.5%). Sample characteristics of participants can be found in Table 1. Differences between GPs who already use AI in their practice and those who do not were found only with respect to area of settlement, indicating that GPs in rural areas were less likely to use AI in their practice (Table 1).

**Table 1.** Sample characteristics of participating GPs (*n* = 154)

	Total *n* =154	Use of AI in private *n* = 144	Use of AI in practice *n* = 70	No use of AI in practice *n* = 77	Statistics: AI versus no AI in practice
**Age, years**	50.6±8.4	50.5±8.4	50.4±9.0	50.8±8.1	n.s.
**Sex**					n.s.
Male	75 (48.7)	70 (48.6)	35 (50.0)	38 (49.4)	
Female	78 (50.6)	73 (50.7)	34 (48.6)	39 (50.6)	
Other	1 (0.6)	1 (0.7)	1 (1.4)	0 (0)	
**Number of years worked as a GP after specialist qualification**	14.2±9.6	14.0±9.5	13.7±9.1	14.8±10.3	n.s.
**Professional status**					n.s.
Established doctor	139 (90.3)	129 (89.6)	61 (87.1)	71 (92.2)	
Employed doctor	15 (9.7)	15 (10.4)	9 (12.9)	6 (7.8)	
**Types of practice**					n.s.
Single practice	101 (65.6)	94 (65.3)	40 (57.1)	56 (72.7)	
Joint practice	23 (14.9)	21 (14.6)	13 (18.6)	9 (11.7)	
Practice sharing	18 (11.7)	17 (11.8)	10 (14.3)	7 (9.1)	
Medical care centre	12 (7.8)	12 (8.3)	7 (10.0)	5 (6.5)	
**Area of settlement**					χ²(3) = 8.474, *P* = 0.035, *V* = 0.243
Rural area	17 (11.3)^a^	15 (10.6)^b^	3 (4.3)	13 (17.8)^c^	
Small town	53 (35.3)^a^	52 (36.9)^b^	30 (42.9)	21 (28.8)^c^	
Medium-sized town	27 (18.0)^a^	27 (19.1)^b^	11 (15.7)	15 (20.5)^c^	
Urban area	53 (35.3)^a^	47 (33.3)^b^	26 (37.1)	24 (32.9)^c^	

^a^ *n *= 150. ^b^ *n *= 141.^c^ *n *= 73. Missing data for Area of settlement, *n* = 4. Data are presented as mean, standard deviations, *n*, and percentage (n/nvalid). n.s.= not significant.

### Current use of AI in GP practices

In our sample, 70 GPs reported currently using AI in their practice, 77 GPs reported not using AI, and seven GPs were uncertain about their AI use (Table 1). Participants who used AI in practice (*n* = 70) predominantly used it for therapy (*n* = 51, 72.9%) and speech recognition and transcription (*n* = 47, 67.1%). Less frequently used AI applications were related to appointment management (*n* = 26, 37.1%), medical billing (*n* = 24, 34.3%), diagnosis (*n* = 23, 32.9%), online consultations (*n* = 20, 28.6%), and patient record management (*n* = 17, 24.3%).

We then asked participants who used AI in practice whether they perceived these AI applications as helpful. The majority of users reported that the AI applications they used were helpful, particularly those used for diagnosis (*n* = 23 of 23 responders, 100%), speech recognition and transcription (*n* = 44 of 47, 93.6%), medical billing, (*n* = 22 of 24, 91.7%), patient record management (*n* = 15 of 17, 88.2%), and appointment management (*n* = 22 of 26, 84.6%). In contrast, AI applications related to therapy (*n* = 36 of 51, 70.6%) and online consultations (*n* = 13 of 20, 65.0%) were considered the least helpful.

### Perceived barriers for using AI in GP practices

The biggest barrier to the use of AI in general practice is a lack of knowledge about suitable AI-based applications and lack of knowledge of how AI works (see all barriers in Table 2).

**Table 2.** Perceived barriers for using AI in GP practices rated on Likert scales across all responders (*n* = 154)

Completely disagree	Rather disagree	Neither agree nor disagree	Rather agree	Completely agree
**Concerns about data security**				
15 (9.7%)	31 (20.1%)	39 (25.3%)	37 (24.0%)	32 (20.8%)
**Concerns about financial aspects or billing**				
17 (11.0%)	48 (31.2%)	31 (20.1%)	39 (25.3%)	19 (12.3%)
**Concerns about reliability**				
7 (4.5%)	37 (24.0%)	44 (28.6%)	43 (27.9%)	23 (14.9%)
**Concerns about whether patients would accept AI**				
8 (5.2%)	48 (31.2%)	52 (33.8%)	35 (22.7%)	11 (7.1%)
**Legal concerns**				
15 (9.7%)	39 (25.3%)	33 (21.4%)	42 (27.3%)	25 (16.2%)
**No knowledge about suitable AI-based apps**				
19 (12.3%)	25 (16.2%)	25 (16.2%)	50 (32.5%)	35 (22.7%)
**No need in daily work**				
25 (16.2%)	48 (31.2%)	45 (29.2%)	19 (12.3%)	17 (11.0%)
**No time to deal with AI**				
26 (16.9%)	34 (22.1%)	45 (29.2%)	33 (21.4%)	16 (10.4%)
**Technical concerns**				
19 (12.3%)	52 (33.8%)	36 (23.4%)	32 (20.8%)	15 (9.7%)
**Technically not possible**				
61 (39.6%)	50 (32.5%)	28 (18.2%)	9 (5.8%)	6 (3.9%)
**Too little knowledge of how AI works**				
10 (6.5%)	32 (20.8%)	35 (22.7%)	49 (31.8%)	28 (18.2%)

Data are presented as *n* and percentage (*n*/nvalid).

**Figure 1.**
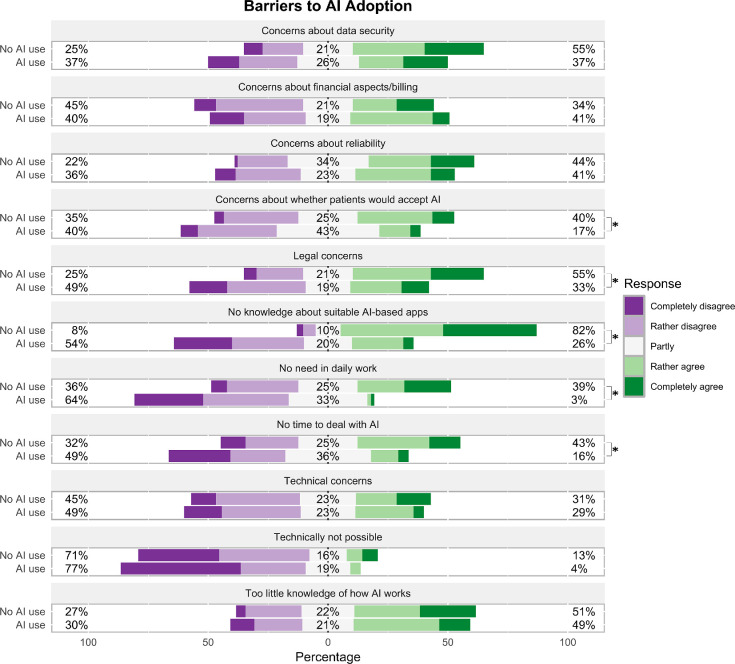
Perceived barriers for using AI in GP practices rated on Likert scales between responders who use (*n* = 70) and do not use AI in their practice (*n* = 77). *P<0.05

We further compared perceived barriers between GPs who do and do not use AI in their practice (Figure 1). Non-users expressed significantly greater legal concerns (χ²[4] = 10.692, *P* = 0.031, *V* = 0.270) and stronger concerns regarding patient acceptance of AI (χ²[4] = 11.101, *P* = 0.024, *V* = 0.275). Additionally, they reported being familiar with fewer AI applications (χ²[4] = 53.668, *P*<0.001, *V* = 0.604), perceived less relevance of AI for their daily work (χ²[4] = 33.707, *P*<0.001, *V* = 0.479), and agreed more on a lack of time to deal with AI (χ²[4] = 15.424, *P* = 0.004, *V* = 0.324). The observed effect sizes span from medium to large, suggesting that the group differences are meaningful in magnitude.

Participants were able to report additional barriers in a free-text field. The most frequently mentioned barriers pertained to technical issues (Table 3). In a separate free-text item, participants were asked to suggest factors that could help reduce these barriers. Most suggestions focused on AI-specific factors such as appropriate pricing, seamless integration into practice software, and improved usability. Governance support would also help to reduce barriers (Table 3).

**Table 3.** GPs’ statements with respect to additional barriers for AI usage (*n* = 36) as well as important aspects to reduce barriers (*n* = 48): content analysis of free-text answers.

Major category	Subcategory	*n* ^a^
*Additional barriers*		
Technical barriers		20
	Defectiveness or immaturity of the applications	9
	AI not compatible with practice software	8
	Poor internet or telephone reception	2
	AI cannot be used during on-call duty	1
Barriers to implementation in practice		13
	AI is too expensive	5
	Difficult to integrate into everyday practice	3
	High demand for staff training	2
	AI is not empathetic and does not understand interpersonal signals	2
	Companies do not develop AI together with practices and do not improve quickly enough	1
Legal barriers		5
	Unclear liability and responsibility	3
	Lack of approval as a medical device	1
	Data protection issues	1
Uncertain benefits		3
	AI does not save time	2
	No benefit for patients	1
Others		3
	High resource consumption	2
	Using your own brain	1
*Barrier reductions*		
AI-related factors		34
	Appropriate cost-benefit balance	9
	Easy integration into practice software	9
	Usability of AI	5
	More transparency about the systems and their capabilities	4
	Reliability of AI applications	4
	Co-development by future users	1
	Being able to try out AI	1
	Reliable and accessible contact persons	1
Governance		20
	Regulated liability or data protection	6
	More time	5
	Reliable overview of certified AI offerings	3
	Relief for GP practices (for example, bureaucracy, financing)	3
	Improved infrastructure	2
	Central and standardised organisation of AI	1
User-related factors		14
	More knowledge (research, training) about AI in GP practices	11
	Recognition that AI cannot replace a doctor, but can support them	2
	Social acceptance	1

^a^ *n* **=** statements in this category.

Some responses were coded into more than one category.

### Perceived needs for using AI in GP practices

The majority of responders (*n* = 83 out of 149 , 55.7%) indicated a need for further training on AI use in GP practices. Those who expressed this need were asked to specify the topics they would like to be trained in. Free-text analysis revealed that participants were primarily interested in learning about the possibilities of using AI, particularly overviews of available and useful applications, AI solutions relevant to practice management, and legal or security-related aspects of AI use in general practice (Table 4).

**Table 4.** GPs’ statements with respect to the need for further training in relation to the use of AI in GP practices (*n* = 59) as well as areas of their daily work they would like to see AI-based applications (*n* = 79): content analysis of free-text answers

Major category	Subcategory	*n* ^a^
*Need for further training in relation to the use of AI in GP practices*		
Possibilities of using AI		31
	Overview of available and useful applications	28
	Potential and future development of AI in GP practices	2
	Possibilities for home visits	1
Practice management		20
	AI for billing services	5
	AI for patient administration	4
	AI for text work	4
	AI for appointment management	2
	Creating and saving medical reports	2
	AI for monitoring medical assistants	1
	AI for telephone assistance	1
	AI for reducing bureaucracy	1
Security or legislation		15
	Legal basis for AI (data protection, liability)	7
	Help with safe establishment or use	6
	Quality of AI applications	2
Medical tasks		11
	Diagnosis	8
	Medication management (drug interactions)	2
	Therapy	1
Technical aspects		9
	Introduction to AI use in GP practices	6
	Technical requirements for AI application in GP practices	1
	Technical understanding behind AI function	1
	AI in interaction with practice software	1
Others		9
	General information on AI	4
	Opportunity to share experiences with colleagues	3
	Research with AI	1
	AI and databases	1
		
*Areas of GPs’ daily work they would like to see AI-based applications*		
Patient care		76
	General diagnostic support	24
	Diagnosis of skin diseases	11
	Medication management	9
	Treatment decision support	8
	Diagnosis of rare diseases	7
	Translation aid	4
	Comparison of repetitive examinations (for example, sonography)	3
	Triage of patients	2
	Evaluation of findings	2
	Incapacity for work in the case of minor illnesses	2
	Patient education	2
	Medical history	2
Practice management		73
	Handling of calls or enquiries regarding appointment requests, (follow-up) prescription or referral requests from patients	19
	Billing	13
	Documentation	10
	Data management (recording of findings, data maintenance, data import and export)	7
	Text recognition or dictation function	6
	Automatic completion of documents from patient file or audio-recording	6
	Answering inquiries from authorities or nursing homes	3
	Check prescribability or protection against financial recourse claims (regress)	3
	Reductions in bureaucracy	3
	Statistics	1
	Reminder function (for necessary interventions or diagnostics)	1
	Organisation of consumables	1
Others		1
	General practice knowledge reference	1

^a^ *n* = statements in this category

In a second free-text field, participants were asked to describe which areas of their daily work they would like to see supported by AI-based applications. The majority of responses related to patient care — particularly diagnostic support in general and specifically for skin and rare diseases — and to practices management, especially the handling of patient enquiries regarding appointments, prescriptions, or referral requests. A complete overview of response categories is provided in Table 4.

### Open free-text field

At the end of the questionnaire, participants were able to provide additional comments on the topic of AI in general practice that had not been addressed in the structured items. Free-text analysis identified three main themes. First, some participants emphasised that AI cannot substitute the interpersonal relationship between physician and patient, and stressed that it should not be used with this intention:

*‘I believe that a personal examination can never be replaced by AI.’* (Female, 61 years)

*‘AI is purely a probability calculation without instinct; all nonverbal and non-physically quantifiable diagnostics are underrepresented or virtually non-existent. What about smell, skin colour, tactile examination? For good medicine, AI is an obstacle — it will only continue to make it more theoretical and less human.’* (Male, 48 years)

Second, some participants voiced concerns about the potential replacement of physicians by AI:

*‘The use of AI will make doctors obsolete — we are abolishing ourselves.’* (Male, 56 years) *‘The goal should not be to compete with the extensive knowledge physicians possess, but rather to open the door to a complementary way of working together. AI cannot and will not replace doctors, but it can enhance their decision-making.’* (Male, 42 years)

Third, participants noted that the initial promises of digitisation were often unfulfilled, leading some to express scepticism about AI’s potential to reduce workload in general practice:

*‘Our recent experiences with the forced implementation of the telematics infrastructure — which cost us far too much time, money, and nerves — do little to inspire confidence that AI, especially if mandated in a centralised manner, could bring any relief to the medical profession.’* (Female, 59 years)

*‘I fundamentally reject AI when its use aims to shift bureaucracy instead of reducing it, or to replace humans for cost reasons! So far, all digital applications have proven to be cost and time traps due to their susceptibility to errors, maintenance demands, hardware upgrades, and the outsourcing of bureaucratic processes from public and private health insurers to practices. Neither medical school, postgraduate training, nor continuing education prepare us for these challenges.’* (Male, 62 years)

## Discussion

### Summary

We assessed GPs’ current use of AI, perceived barriers, and needs related to AI applications in general practice in Germany. We surveyed 154 GPs. AI was applied in some practices (*n* = 70), particularly for tasks such as therapy support, speech recognition and transcription, and administrative processes. These applications were generally perceived as helpful by users, especially for diagnostic support and documentation-related tasks, but less for therapeutic uses.

The results highlight a knowledge gap as a major barrier to AI implementation, both in terms of how AI works and awareness of suitable applications. Data security, legal, and reliability concerns were additional barriers. Compared with AI users, non-users expressed greater legal concerns, more concerns about patient acceptance, less familiarity with suitable AI applications, and perceived AI as less relevant to their daily work.

To reduce barriers to AI adoption, participants emphasised a favourable cost–benefit ratio of AI applications and facilitating seamless integration into practice software. The majority of responders also expressed a need for further training on the use of AI in general practice, particularly overviews of available and useful applications.

GPs primarily expressed interest in utilising AI for both patient care and practice management. They envisioned its use in diagnostic support, handling patient inquiries and appointment scheduling, processing prescription and referral requests, and improving billing and documentation tasks. Notably, GPs recognised the potential of AI to facilitate their daily work, but emphasised the importance of maintaining human elements in care. They voiced scepticism about the potential of AI to truly alleviate workload, reflecting broader concerns about the trajectory of digitalisation in health care.

### Strengths and limitations

A strength of the present study is the heterogeneous sample, with participants representing a broad range of ages, genders, and practice settings. Although the response rate was low, the absolute number of responders is relatively high compared with similar studies. Importantly, this study provides an integrated perspective on the current use, perceived barriers, and specific needs of GPs regarding the adoption of AI. These findings offer insights for healthcare stakeholders to better support GPs and guide the future development of AI applications tailored to primary care.

The low response rate may introduce selection bias, as it is likely that participation was skewed towards individuals already interested in AI. Those with strong reservations or opposition to AI may have been less inclined to engage with the survey. In addition, we used a broad definition of AI, which may have led to some misclassification of responders’ experiences or knowledge. The cross-sectional design precludes any conclusions about causal relationships. Finally, all data were collected via self-report, which may be subject to response biases such as social desirability or recall bias.

### Comparison with existing literature

Demographic differences in AI usage were found to be small in our and in other studies.^12^ In the context of current AI use among GPs, therapy emerged as the most frequently reported application area. Despite this, satisfaction with AI in therapy was lower than in other domains. One possible explanation is the association with digital health applications (DiGAs), which were provided as examples of AI in therapy within the survey. Previous studies have shown that GPs have a generally critical stance towards DiGAs usefulness.^13^ This scepticism may contribute to the reported dissatisfaction, suggesting that while AI tools are being used, their perceived value in therapeutic practice remains limited.

Barriers stated in our study support the existing literature,^5,9,14,15^ indicating that a lack of knowledge is a central barrier in AI implementation in primary care. Particularly, a study identified considerable gaps in knowledge related to the development, implementation, and evaluation of AI tools in primary care.^5^ GPs in our study also expressed concerns regarding data protection, legal safeguards, and the reliability of AI systems. These findings are consistent with a study conducted in Germany, which reported widespread concerns regarding the reliability, accuracy, and trustworthiness of AI systems, as well as fears that AI might undermine professional autonomy.^9^ A similar study of GPs in the UK found that most of their concerns were related to clinical liability, accuracy, and patient safety.^16^ To reduce barriers, the need for mature AI tools to meet the practical needs of healthcare providers^5^ and their alignment with regulatory frameworks to enhance acceptance and accountability^14^ was reported elsewhere.

Concerns about being replaced by AI and the potential impact on the doctor–patient relationship have also been reported in other studies.^9,14–16^ These issues should be proactively addressed by policymakers and technology developers to ensure that AI is promoted explicitly as a supportive tool, rather than a substitute for physicians.

### Implications for research and practice

Consistent with previous research, our findings underscore the need for ongoing education on the use of AI in general practice. Professional associations can use these insights to develop targeted training programmes that address GPs’ specific needs and concerns. Tailored educational initiatives may enhance GPs’ competence in applying AI tools and help reduce barriers to adoption, particularly among current non-users.

In addition, trade associations may advocate for clearer legal and regulatory frameworks and communicate these needs to policymakers. Issues relating to data security and legal responsibility must be addressed through appropriate legislation to foster trust and usability.

Finally, GPs’ concerns about the impact of AI on the doctor–patient relationship should be taken seriously. Further research is needed to explore these concerns and to develop user-centred implementation strategies. In addition, we recommend that future studies include other ambulatory specialist and hospital-based physician groups to provide a more comprehensive understanding of AI adoption, perceived barriers, and training needs across different healthcare settings. It is essential to clearly communicate the role of AI as a supportive tool, rather than a replacement for physicians, in order to maintain trust in the profession and promote responsible innovation.

## Data

Data will be made available on request.
